# Survival of the Likeliest?

**DOI:** 10.1371/journal.pbio.0050142

**Published:** 2007-05-15

**Authors:** John Whitfield

## Abstract

This feature explores how the laws of thermodynamics to explain natural selection and life itself.

*Using the laws of thermodynamics to explain natural selection—and life itself*

At first glance, life and the laws of thermodynamics seem to be at loggerheads. Most glaringly, the second law states that over time, any system will tend to the maximum level of entropy, meaning the minimum level of order and useful energy. Open a bottle of perfume in a closed room, and eventually the pool of scent will become a smelly cloud. Organisms do their damnedest to avoid the smelly cloud of equilibrium, otherwise known as death, and a common argument of anti-evolutionists is that the universe's tendency toward disorder means that natural selection cannot make living things more complex. The usual counter to this argument is that organisms maintain internal order and build complexity by exporting entropy—importing energy in one form, and radiating it out in another, higher-entropy form. One of the first physicists to ponder these questions, Erwin Schrödinger, described food as negative entropy: “The essential thing in metabolism is that the organism succeeds in freeing itself from all the entropy it cannot help producing while alive.”[1]

Darwinian selection….isn't the only thing that can create order.

But recently, some physicists have gone beyond this and argued that living things belong to a whole class of complex and orderly systems that exist not despite the second law of thermodynamics, but because of it. They argue that our view of evolution, and of life itself, should likewise be based in thermodynamics and what these physical laws say about flows of energy and matter. Darwinian selection, these researchers point out, isn't the only thing that can create order. Throughout the universe, the interaction of energy and matter brings regular structures—be they stars, crystals, eddies in fluids, or weather systems in atmospheres—into being. Living things are the most complex and orderly systems known; could they be part of the same phenomenon? And could the process that brings them about—natural selection, driven by competition between organisms—be ultimately explicable in thermodynamic terms?

Eric Smith, a theoretical physicist at the Santa Fe Institute in New Mexico, certainly thinks so. “Darwinian competition and selection are not unique processes,” he says. “They're a complicated version of more fundamental chemical competitive exclusion.” In a paper published last year [2], Smith and his colleagues argued that natural selection is a highly sophisticated version of a physical process called self-organization, the still poorly understood means by which energy plus matter can equal order.

Such orderly, self-organized systems are like engines designed to level out energy gradients—while they persist, they produce more entropy, more quickly, than a disordered mishmash of molecules. Weather systems, for example, transport heat from the tropics toward the poles far more quickly than a homogeneous, static atmosphere would. Life does the same thing, Smith points out. Indeed, he believes that this might have been the reason for its origin—that, under the conditions on early Earth, life was the best way to release the build-up of geothermal energy and an inevitable consequence of that energy [3]. Once biochemistry had got going, subsequent chemical and Darwinian selection would each favor the systems best at dissipating Earth's pent-up energy, whether geothermal or, following the invention of photosynthesis, solar.

It has long been suggested that self-organized systems do not just level out energy gradients more quickly than disordered ones do, they do it as quickly as possible. Models that assume maximum entropy production (MEP) make good predictions about the climates of Earth [4] and Saturn's moon Titan [5] and about the growth of crystals in solutions [6]. But until recently, MEP was just an assumption—there was no mechanism or theory to explain why such systems should tend to this state. Classical thermodynamics is no help— it explains entropy only in closed systems, with no energy going in or coming out. It says nothing about how much entropy open, nonequilibrium systems, such as organisms, ought to produce.

In physics, to speak of natural selection is to ask, among all possible states, which is the one that nature selects.

Roderick Dewar, a theoretical physicist and ecosystem modeler working at the French agricultural research agency's centre in Bordeaux, believes he has crossed this hurdle. Using information theory, a branch of mathematics that can reformulate the laws of thermodynamics (see the Box), Dewar has shown that MEP is the most probable behavior of an open, nonequilibrium system made up of many interacting elements, provided that system is free to “choose” its state and not subject to any strong external forces [7]. The large-scale state of MEP represents the largest proportion of the countless possible arrangements of the system's microscopic parts, regardless of what those parts are up to.

Natural selection in biology could work the same way, Dewar thinks: “In physics, to speak of natural selection is to ask, among all possible states, which is the one that nature selects.” This, he points out, is a question of probability. “The state that nature selects is the one that can be realized in more ways than any other. Biologists don't think like that, but I want to entertain the hypothesis that natural selection in biology works the same way, and see where that gets us.”

Adding life to physical systems certainly increases entropy production. A pond full of plankton or a patch of grass absorbs more of the Sun's energy, and so produces more entropy, than a sterile pool or bare rock. Earth turns sunlight into microwave radiation, closer to equilibrium with the background glow of the Universe, more efficiently than either Mars or Venus. Ecological processes such as succession, where a grassland becomes a forest, also increase entropy production (Figure 1). And over evolutionary time, organisms tend to get better at grabbing energy—witness our own species, which now uses about 40% of the energy in sunlight, and is busy releasing the energy trapped in fossil fuels and converting it into entropy. But can such processes be explained as part of a tendency towards maximum entropy production, rather than a Darwinian competition to leave descendents? The key question is whether living things are really free to arrive at a state of MEP, or whether natural selection is precisely the sort of force that can override such a process.

**Figure 1:**
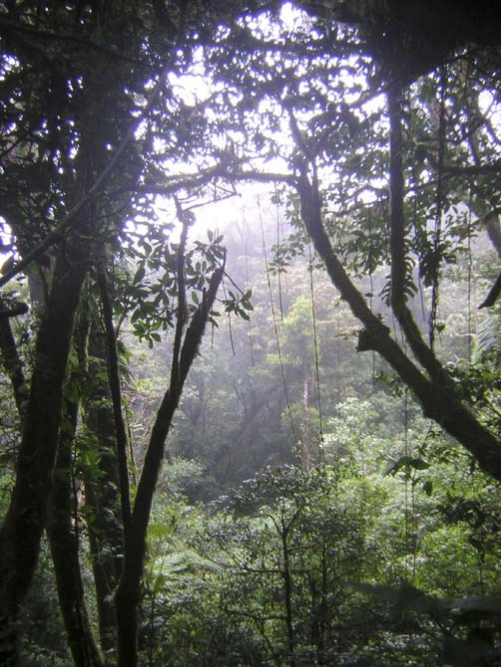
Entropy and biodiversity are mathematically equivalent, making tropical forests the most entropic environments on Earth. (Photograph: John Whitfield)

It seems odd that natural selection could be not survival of the fittest, but arrival at the likeliest, but Dewar thinks just that. Recently, for example, he and his colleagues showed that the structure and workings of the ATP synthase enzyme are predictable using MEP theory [8]—that being an efficient generator of cellular fuel and an efficient leveler of energy gradients are one and the same. In general, Dewar wants to show that biological processes that maximize the rate at which energy is captured, or chemicals transported from one spot to another can be explained from the viewpoint of statistical mechanics—the area of physics that explains how predictable behavior emerges from large groups of unpredictable elements. “Statistical theory would say that the molecules choose the state of maximum flux because that is the most probable way for the molecules in the system to arrange themselves,” says Dewar. “Perhaps they're selecting that state simply because it's the most probable one.” And unlike the conventional view of evolution, this approach allows one to make quantitative predictions of how living things should work. “Darwinian selection is a hypothesis that's quite difficult to quantify,” says Dewar. “It doesn't really come up with numbers.”

A few biologists are beginning to use MEP. “Dewar's proof is brilliant and potentially of enormous consequence for many areas of science,” says ecologist John Harte of the University of California, Berkeley. One such area could be ecology, he adds: “Very preliminary initial explorations of its implications for understanding food webs, material and energy allocation within organisms, and climate-ecosystem interactions are encouraging.”

**Entropy**

Entropy is a powerful but slippery concept. One reason for both its power and its slipperiness is that several different branches of physics have been able to formulate the second law of thermodynamics independently. This has meant that other fields, such as computing and ecology, can use the concept of entropy, and so entropy takes rather different forms in different systems.

In thermodynamics, entropy is uselessness. An energy gradient, such as a difference in temperature, can be used to do work. But as the gradient levels out, the energy is transformed into useless heat in equilibrium with its surroundings. In statistical mechanics, a system's entropy is the number of possible arrangements of all its microscopic states that yield any particular macroscopic state. Maximum entropy is the most probable, and most disordered state. For example, for 1,000 flipped coins, the most likely, and also the most entropic state, is 500 heads and 500 tails. This form of entropy has also been called “mixedupness”: a far greater number of molecular arrangements yield a cup of white coffee than yield a black coffee with a layer of milk sitting on top of it.

In information theory, entropy is uncertainty. The most entropic systems are those in which one is least certain what is coming next. In a very orderly message, such as a string of identical letters, the next letter is predictable. Such a system has no entropy. A string of random letters is very noisy, carries no information, and has the maximum possible entropy. This formulation of entropy was devised by the mathematician Claude Shannon, who also gave his name to a measure of biodiversity, the Shannon index. This index expressed how evenly individuals are distributed within a number of categories. The more categories, and the more equal the number of individuals in each, the greater the biodiversity; this is mathematically equivalent to a measure of entropy. In the most diverse ecosystems, a naturalist has little or no idea what species she will find next.

Another physicist trying to use thermodynamics to predict the details of biological structures is Adrian Bejan, an engineer at Duke University in Durham, North Carolina. Rather than thinking about a system's microscopic elements, Bejan has devised what he calls the “constructal law” [9], a description of how energy and matter flow in physical networks such as river basins and biological networks such as blood vessels. Bejan's constructal law states that for a flow system to persist (i.e., live), it must over time provide easier access to the currents that flow through it—it must come to do more with less, in other words. In the process, it minimizes the amount of fuel used and maximizes the amount of entropy produced for each unit of fuel burnt.

Evolution, Bejan believes, has been a process whereby structures have remodeled themselves so that energy and matter flow through them as quickly and efficiently as possible [10]. Better flow structures—be they animals or river networks—have replaced poorer. This, says Bejan, is a second arrow of time to set alongside the second law's drive towards disorder. The patterns of animal locomotion, he has argued, in particular how animals' stride or flapping frequency changes with body size, is such that animals flow over the surface of Earth as easily as possible [11]. “Given the freedom to morph, a flow system will renew itself to construct easier flow structures,” says Bejan. “The way that animal mass flows over the earth follows the same principle as the way the water of the Amazon flows across the landscape.”

Dewar is not so sure, arguing that the constructal law deals with phenomena, rather than causes. “Rather than explaining why systems should adopt optimal behaviors, Bejan proposes that they do, and then shows that this is realistic,” he says. “It's not very clear what is being maximized—it seems to be anything he can think of.” For his part, Bejan thinks that Dewar's focus on a system's smallest elements is unnecessary: “One doesn't need to go into the microscopic to account for the macroscopic.”

The patterns of animal locomotion… is such that animals flow over the surface of the earth as easily as possible.

Besides these differences among physicists, many biologists, not surprisingly, resist attempts to colonize their discipline. The late Ernst Mayr argued that processes such as reproduction, natural selection, and inheritance have no equivalence in, and are not reducible to, physics, and that biology should be seen as an autonomous science, separate and equal [12]. (Although not everyone in the pantheon of biology thought this way: Francis Crick wrote that the “ultimate aim” of biology should be to explain itself in terms of chemistry and physics [13].)

Lloyd Demetrius, a mathematical biologist at Harvard University, is certainly no physics-phobe. Taking the statistical mechanics–based approach of treating organisms as if they were molecules in a gas, he has formulated a quantity that he calls “evolutionary entropy” [14]. This is mathematically equivalent to thermodynamic entropy, but instead of physical disorder, it describes the age range over which an organism reproduces. Over long periods of evolution, Demetrius expects natural selection to increase this, because organisms that can reproduce over a longer period are better at dealing with limited resources and unpredictable environments.

But evolutionary entropy is not maximized in Demetrius' models, nor does it inevitably increase through time. There are, he says, fundamental differences between thermodynamic processes and natural selection, and biological and physical selection become one only at the molecular level. Any more complicated living system is subject to forces that do not operate in purely physical systems. “In an evolutionary process you have analogues to physical laws, but the mechanisms are quite different,” says Demetrius. “As you go from molecules to cells and higher organisms, selection involves replication, and there's no replication in physics. It's what distinguishes the living from the dead.”

Perhaps in another hundred years, no one will think that we need one set of theories for biology and another for physics.

For the physicists struck by the parallels between self-organized and living systems, however, even this distinction is not as clear-cut as it might seem. “There's a continuum between life and non-life, and the black and white distinction between the two has to be minimized,” says Charles Lineweaver, an astronomer and astrobiologist at the Australian National University in Canberra.

Lineweaver has proposed a category of objects that he calls “far from equilibrium dissipative systems,” which includes all systems that dissipate energy in the process of maintaining themselves in an ordered, non-equilibrium state—including galaxies and hurricanes, as well as plants and animals (Figure 2). It's possible, he believes, that all such systems might be usefully described as alive, and that life should be defined in thermodynamic terms. “As a physicist I'm looking for physics-based definitions of life,” says Lineweaver. ”Biologists are unduly myopic when it comes to this.”

Lineweaver also thinks the replication question is a red herring. To think that life has to store the instructions for its reproduction internally is, he says, arbitrary. The formation of stars, he points out, depends on the preceding generation of stars releasing elements and modifying the gravity of their environment. Everything depends on its environment for energy and materials; where the information is stored doesn't matter. “Shifting the definition of life to a thermodynamic, one removes the mystique from life, in the same way that Darwin said: ‘Hey, we're another type of animal’,” Lineweaver says.

**Figure 2:**
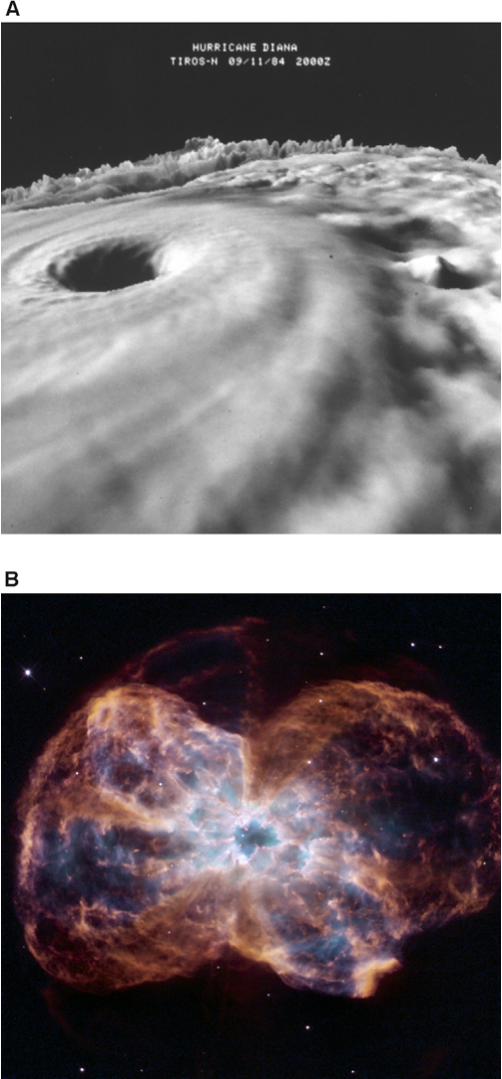
If processes underlying life are explained as a tendency towards maximum entropy production, systems such as galaxies and hurricanes might be described as alive. (A) Three dimensional cloud-top image of Hurricane Diana as it was strengthening from a Category III storm to a Category IV storm. Publication of the National Oceanic and Atmospheric Administration (NOAA), NOAA Central Library (Image ID: spac0289, NOAA in Space Collection) (B). The colorful demise of a sun-like star. [Photo credit: NASA, ESA, and K. Noll (STScI); acknowledgment: The Hubble Heritage Team (STScI/AURA)]

One hundred years ago, one of the hottest debates in biology concerned vitalism—whether living things were made from the same chemicals as inanimate matter, and whether they were animated by a “vital force” unique to biological systems, or obeyed the same laws of physics as dead matter. A century on, we know that living things and dead things are made from the same stuff, and subject to the same forces. Perhaps in another hundred years, no one will think that we need one set of theories for biology and another for physics.

“We should definitely look for common principles,” says Dewar. “If such principles exist, we ought to be able to fuse natural selection in biology with natural selection in physics. Animals competing and dying are ultimately molecular processes that take place under the constraints of energy and resources.”
